# Isolation and characterization of high affinity and highly stable anti-Chikungunya virus antibodies using ALTHEA Gold Libraries™

**DOI:** 10.1186/s12879-021-06717-0

**Published:** 2021-10-30

**Authors:** M. Pedraza-Escalona, O. Guzmán-Bringas, H. I. Arrieta-Oliva, K. Gómez-Castellano, J. Salinas-Trujano, J. Torres-Flores, J. C. Muñoz-Herrera, R. Camacho-Sandoval, P. Contreras-Pineda, R. Chacón-Salinas, S. M. Pérez-Tapia, J. C. Almagro

**Affiliations:** 1 CONACyT-Unidad de Desarrollo e Investigación en Bioprocesos (UDIBI), Escuela Nacional de Ciencias Biológicas, Instituto Politécnico Nacional, Mexico City, México; 2 Unidad de Desarrollo e Investigación en Bioprocesos (UDIBI), Escuela Nacional de Ciencias Biológicas, Instituto Politécnico Nacional, Mexico City, México; 3 Laboratorio Nacional para Servicios Especializados de Investigación, Desarrollo e Innovación (I+D+i) para Farmoquímicos y Biotecnológicos, LANSEIDI-FarBiotec-CONACyT, Mexico City, México; 4 Departamento de Inmunología, Escuela Nacional de Ciencias Biológicas, Instituto Politécnico Nacional (ENCB-IPN), México City, México; 5 GlobalBio, Inc, 320 Concord Ave., 02138 Cambridge, MA USA

**Keywords:** Chikungunya virus, Venezuelan equine encephalitis Virus, Diagnostic, Human Antibodies, Next-Generation Sequencing, Phage display

## Abstract

**Background:**

More than 3 million infections were attributed to Chikungunya virus (CHIKV) in the 2014–2016 outbreak in Mexico, Central and South America, with over 500 deaths directly or indirectly related to this viral disease. CHIKV outbreaks are recurrent and no vaccine nor approved therapeutics exist to prevent or treat CHIKV infection. Reliable and robust diagnostic methods are thus critical to control future CHIKV outbreaks. Direct CHIKV detection in serum samples via highly specific and high affinity anti-CHIKV antibodies has shown to be an early and effective clinical diagnosis.

**Methods:**

To isolate highly specific and high affinity anti-CHIKV, Chikungunya virions were isolated from serum of a patient in Veracruz, México. After purification and characterization via electron microscopy, SDS-PAGE and binding to well-characterized anti-CHIKV antibodies, UV-inactivated particles were utilized as selector in a solid-phase panning in combination with ALTHEA Gold Libraries™, as source of antibodies. The screening was based on ELISA and Next-Generation Sequencing.

**Results:**

The CHIKV isolate showed the typical morphology of the virus. Protein bands in the SDS-PAGE were consistent with the size of CHIKV capsid proteins. UV-inactivated CHIKV particles bound tightly the control antibodies. The lead antibodies here obtained, on the other hand, showed high expression yield, > 95% monomeric content after a single-step Protein A purification, and importantly, had a thermal stability above 75 °C. Most of the antibodies recognized linear epitopes on E2, including the highest affinity antibody called C7. A sandwich ELISA implemented with C7 and a potent neutralizing antibody isolated elsewhere, also specific for E2 but recognizing a discontinuous epitope, showed a dynamic range of 0.2–40.0 mg/mL of UV-inactivated CHIKV purified preparation. The number of CHIKV particles estimated based on the concentration of E2 in the extract suggested that the assay could detect clinically meaningful amounts of CHIKV in serum.

**Conclusions:**

The newly discovered antibodies offer valuable tools for characterization of CHIKV isolates. Therefore, the strategy here followed using whole viral particles and ALTHEA Gold Libraries^™^ could expedite the discovery and development of antibodies for detection and control of emergent and quickly spreading viral outbreaks.

**Supplementary Information:**

The online version contains supplementary material available at 10.1186/s12879-021-06717-0.

## Background

Chikungunya fever is a mosquito-borne viral disease caused by Chikungunya virus (CHIKV). Its name derives from a Makonde word translated as ‘disease that bends up the joints’, which describes the posture of afflicted individuals feeling severe joint pain or arthralgia [1]. Other symptoms include abrupt fever, muscle pain, headache, nausea, fatigue, and rash. These symptoms are often mild, and the infection may go unrecognized or not accurately diagnosed, in regions where other infections caused by Dengue virus (DENV) and Zika virus (ZIKV) occur.

CHIKV was first isolated in 1952 in Tanzania [1] and reemerged within a span of several years in South East Africa and Asia [2]. In December 2013, CHIKV reached the Americas [3], first reported on St. Martin in the Caribbean and within weeks in neighboring islands. The first CHIKV report in Mexico occurred in May 2014 from a patient in Jalisco, who showed CHIKV infection symptoms after a trip to the Caribbean region [4]. Other reports followed in October 2014 in Chiapas [5, 6]. By 2015, over eleven thousand cases occurred mostly in the southern region of the country [7] where warm and humid zones are amenable to mosquito growth. Overall, more than three million CHIKV infections have been reported in Mexico, Central and South America, with over 500 deaths directly or indirectly related to this viral disease [8].

While CHIKV infection is not currently considered a public health concern, with only three cases reported by mid-2019 (www.gob.mx/salud/acciones-y-programas/chikungunya-informacion-relevante), chikungunya fever outbreaks are recurrent [2, 9]. Some of the factors contributing to such a recurrence are an inadequate disease surveillance and underdiagnosis, virus evolution and mutation, new generations of individuals with no immunity to the virus, supply chains globalization, travelling, and climate change. Since no approved vaccine nor antiviral CHIKV therapeutics exist, early detection and appropriate disease management are the only tools at the disposal of the scientists, health care community and governments to control future CHIKV outbreaks and reduce their devastating social and economic impacts.

Three CHIKV major lineages have been described thus far: West African (WA); East, Central, and South African (ECSA); and Asian [10, 11]. In addition, an Indian Ocean (IOL) sub lineage emerged within the ECSA and the Asian/American sub lineage emerged within the Asian lineages. The surface of CHIKV particles contains two main structural glycoproteins, E1 and E2, which have been considered targets in the development of diagnosis tools [12], therapeutic antibodies [13], and vaccines [14]. E2 protein shows the highest percentage of amino acid variation among the lineages and strains, impacting in some cases the interaction with the host receptor and hence modifying CHIKV clinical manifestations, virulence and even its epidemiology [15]. These changes could also modify the interaction with specific antibodies, limiting the use of such antibodies as diagnostic, therapeutic or prophylactic drugs.

Detection methods of CHIKV infection are based on virus isolation, reverse transcriptase polymerase chain reaction (RT-PCR) [16] and IgM/IgG ELISA [17]. Since virus isolation requires highly secure (BSL-3 or BSL-2 plus) facilities, PCR-based methods are mostly used for early and accurate diagnosis. Nonetheless, relatively high cost of the PCR-based assay and the requirement of a thermal cycler to conduct the test limit its application in the field and rural regions where outbreaks often occur. Immunochemical methods such as IgM/IgG ELISA can be applied everywhere and are cost-effective, but it takes around a week for the patient to develop an antibody response. Hence, IgM/IgG ELISA as a diagnosis tool is not effective in early stages of the infection.

Alternatively, direct CHIKV detection in serum has been reported to be an early and reliable clinical diagnosis as well as an effective surveillance of CHIKV [12, 18, 19]. Some of the tests relies on anti-CHIKV monoclonal antibodies obtained *via* hybridoma technology after immunization with inactivated CHIKV particles or recombinant proteins derived from the virus [12, 20]. Virus-like particles (VLPs) have also been used as selector combined with immune antibody libraries or B-cell sorting as source of human anti-CHIKV antibodies [13, 21]. Due to the exquisite specificity of the antibodies however, one of the potential limitations of direct CHIKV detection in serum samples is the differential sensitivity and specificity of the tests for detection of diverse CHIKV lineages. For instance, it has been reported [22] that a test with high sensitivity and specificity for clinical samples known to contain ECSA-genotype has low sensitivity with clinical samples obtained from individuals infected with the Asian genotype [23]. Obviously, surveillance strategies based on results of tests with low sensitivity for a given CHIKV lineage would be misleading when applied in regions where the outbreak is caused by such a lineage.

Here, with the goal of generating anti-CHIKV antibodies with potential application in the early diagnostic and CHIKV disease management in Mexico, Chikungunya virions were purified from a serum sample from a patient from Veracruz, México, during the 2015 CHIKV outbreak. The integrity of the Chikungunya virions was assessed by structural methods such as electron microscopy as well as functional assays such as binding to diverse well-characterized anti-CHIKV monoclonal antibodies [13, 21]. We also determined the whole genome sequence of this CHIKV isolate and a comparison with several strains of different lineages described for CHIKV, e.g., WA, ECSA and IOL lineages was made.

Once qualified, UV-inactivated CHIKV particles were used as selectors to discover high affinity and highly stable antibodies from ALTHEA Gold Libraries™ [24, 25]. The specificity of the antibodies was assessed by ELISA against mosquito-transmitted viruses from Venezuelan equine encephalitis virus (VEEV), which belongs to the *alphavirus genus* of the Togaviridae family, and DENV (DENV-1 and DENV-2) and ZIKV, which belong to *Flavivirus genus* of the Flaviviridae family. Since DENV and ZIKV could co-exist with CHIKV and generate similar symptoms, it makes difficult the differential diagnostic of CHIKV infection. Finally, a sandwich ELISA with the best antibody, combined with a potent neutralizing antibody reported elsewhere [13], was carry out. The results indicate that the newly discovered antibodies and the prototype ELISA developed herein could be valuable and effective tools for CHIKV disease surveillance.

## Methods

### Cells and viruses

All the virus strains used in this work were propagated and titrated in Vero cells (ATCC CCL-81) using standard plaque assays. Vero cells were grown in complete Eagle’s Minimal Essential Medium (ATCC 30-2003) supplemented with 10 % FBS. The CHIKV-033 isolate, was kindly donated by Dra. Rosalia Lira from Unidad de Investigación Médica en Enfermedades Infecciosas y Parasitarias, IMSS, México. The VEEV strain TC-83, derived from ATCC VR-1249TM, was directly amplified in Vero cells from the VEEV vaccine (EQUIVAC TC-83, Productora Nacional de Productos Veterinarios, Mexico). The ZIKV isolate (Yucatán, México), the DENV-1 strain Puerto Rico/PR159-S1/1969 and the DENV-2 strain YUC17438 were kindly donated by Dr. Ma Isabel Salazar Sánchez from the Escuela Nacional de Ciencias Biológicas, National Polytechnic Institute, México.

### Virus assays

The alphaviruses (CHIKV-033 and VEEV strain TC-83) and flaviviruses (ZIKV, DENV-1 and DENV-2) were routinely propagated in our laboratory. Briefly, Vero cells were grown in 75 cm^2^ flasks until a confluence of 80% was reached. Afterwards, the growth media was removed, and the cells were infected with alphaviruses at a MOI of 0.001, or Flaviviruses at a MOI of 0.1 using 1 mL of viral inoculum per 75 cm^2^ bottle. After an adsorption period of 2 h, the viral inoculum was removed and maintenance media (EMEM supplemented with 3% FBS) was added. The infection was left to proceed for 3 days for the alphaviruses, and 4 days for the Flavivirus ZIKA and 7 days for DENV-1 and DENV-2, afterwards the infected cultures were frozen and thawed once, and the viral lysates were supplemented with 20% FBS, clarified and kept at −80 ºC until further use.

### CHIKV purification

Before virus purification, CHIKV-033 lysates were inactivated by three cycles of UV light for periods of 30 min. The inactivation of final preparations was verified using standard plaque assays in accordance with Warter and co-workers [21]. CHIKV-033 particles were precipitated from the inactivated viral lysates with cold PEG-NaCl at least 12 h. The mixture was centrifugated at 3000xg for 30 min at 4 ºC. The precipitated CHIKV particles were resuspended in TNE buffer (50 mM Tris–HCl, 100 mM NaCl, 0.1 mM EDTA, pH 7.4) at 4 °C. The CHIKV stock from PEG precipitation (25 mL) was then layered over 10 mL of a 60−30 % (w/v) sucrose step gradient prepared in ultracentrifuge tubes (Beckman Coulter, Cat No. 349622) on ice. Ultracentrifugation was carried out at 121,000×*g* for 3 h at 4 °C using a Beckman SW-28 rotor. After centrifugation, the CHIKV particles were observed as a white band above the 60% (w/v) sucrose layer. The particles were collected using a Pasteur pipette and diluted in TNE buffer. To pellet down the virus, 10 mL of sample with TNE buffer was layered over 2 mL of 30% (w/v) sucrose to a 12 mL ultracentrifuge. A second ultracentrifugation step was carried out at 281,000×*g* for 1 h at 4 °C using a Beckman SW41Ti rotor. The supernatant was removed, and the pellet was resuspended with 200 µL of TNE buffer.

### Transmission electron microscopy (TEM)

Negative staining TEM was used to analyze the shape and size of the purified viral particles. Chikungunya particles (10 µL) were fixed for 1 min on copper grids coated with Formvar-carbon (Electron Microscopy Sciences, Cat No. CF200-Cu) and stained with 2.5% (v/v) uranyl acetate for 15 s. After air dry, samples were observed in a Zeiss Libra 120 transmission electron microscope at 80 kV. The images of the CHIKV were displayed using ImageJ software (National Institutes of Health, Bethesda, MD).

### Control antibodies

Three control antibodies were used in this work: two human antibodies (4J21 and 4N12) reported by [13] and another antibody (5F10) reported by [21]. 4J21 and 4N12 were cloned as human IgG1/kappa molecules from the V regions disclosed in the patent WO 2016/168,417 and Protein-A purified in house—See IgG conversion below. 5F10 was purchased at Novus Biologicals (Clone E26D9.02, Cat No. DDX9 100P-100).

### Direct binding ELISA

Nunc MaxiSorp™ flat-bottom plates (Thermo Scientific; Cat No. 44-2404-21) were coated with purified CHIKV particles at 3 µg/ml in PBS overnight at 4 °C. After blocking with 3% skim milk prepared in PBS (MPBS) for 1 h, IPTG-induced cultures, purified scFvs or IgGs in MPBS were added to the wells in 3-fold serially dilutions and incubated for 1 h at 37 °C. Bound samples were detected with recombinant Protein-A conjugated to HRP (1:8000, Invitrogen, Thermo Fisher Scientific; Cat No. 101123). The assay was revealed with TMB Substrate Reagent Set (BD OptEIA, BD Biosciences; Cat No. 555214). The reaction was stopped by the addition of 1 M phosphoric acid. The absorbance was read at 450/570 nm using an automated BioTek´s 2 microplate reader.

### ALTHEA Gold Libraries™ and panning using inactivated CHIKV particles

ALTHEA Gold Libraries™ consist of two semisynthetic libraries built with synthetic human germline genes combined with natural human HCDR3/J_H_ (H3J) fragments obtained from peripheral blood mononuclear cells (PBMCs) of a large pool of 180 donors. One of the libraries, called SL1, was built with a V_L_ scaffold assembled with the human IGKV3-20*01 germline gene combined with the human IGJV4*01 joining region. The other library, called SL2, was made with a V_L_ scaffold built with the human IGKV4-01*01 germline gene, also combined with the IGJV4*01 germline gene. Both libraries have a universal V_H_ scaffold, which was partially built with the human IGHV3-23*01 germline gene. SL1 has a short LCDR1, whereas SL2 has a long one. By changing the length of LCDR1 from short to long, antibodies alter the preference to bind protein or peptide targets, respectively [26, 27]. Therefore, by using the library with the proper V_L_ scaffold, antibodies against protein or peptide targets can be selected. When used in combination, SL1 and SL2 would potentially produce antibodies that bind diverse epitopes on a given target.

Selection of anti-CHIKV antibodies was performed in solid phase. Nunc MaxiSorp™ flat-bottom plates (Thermo Scientific; Cat No. 44-2404-21) were coated with purified CHIKV-033 at a concentration of 5 µg/mL in PBS. Aliquots of 10^11^–10^12^ cfu from ALTHEA Gold Libraries™, covering 10 to 100 times the initial library diversity, were used in the first round of panning. Subsequent rounds were performed with the output of the previous round at 10^12^ cfu.

### Screening for positive and unique anti-CHIKV scFvs

Soluble scFvs were induced with IPTG (1 mM) and tested in plates with CHIKV or BSA as negative control. As reporter reagent, Protein-A/HRP (Thermo Fisher; Cat No. 101,123) was used. PCR of the positive clones were submitted to Sanger sequencing and unique clones were expressed as soluble scFvs, purified using Protein-A and re-tested for binding to CHIKV and BSA in direct ELISA assays.

### IgG conversion

The scFvs identified in the ELISA-based screening were converted to human IgG1 *via* PCR of the V regions and cloning in TGEX expression vectors (Antibody Design Labs, Inc). The V regions of the sequences identified by NGS were PCR amplified with a reverse primer matching the HCDR3 sequences and a universal forward primer hybridizing in the leader peptide. The IgGs were expressed in HEK 293 cells (ATCC CRL-3216) by co-transfection of plasmid DNA containing the heavy and light chains. After a 4-day incubation, the supernatants containing the IgGs were harvested, filtrated and purified using Protein A MabSelect SuRe column (5 mL, GE Healthcare). The IgGs were captured in PBS (20 mM, 150 mM NaCl, pH 7.4) and eluted with 20 mM citrate buffer pH 3.5. The monomeric content of the purified IgGs was estimated by UPLC BEH200 150 mm Size Exclusion Chromatography (SEC) columns (Waters). The integrity of the IgG was evaluated by SDS-PAGE and Mass Spectrometry (Intact Mass).

### Specificity assay

The cross-reactivity experiment was performed in MaxiSorp 96-well plates coated with 100 µL of purified particles from *alphavirus* genus: CHIKV (3 µg/mL) and VEEV (1 µg/mL) in PBS, pH 7.4, for 16 h at 4 °C. Additionally, inactivated flaviviruses lysates from ZIKV, DENV-1 and − 2 (30 µg/mL) were used. The plates were then incubated with MPBS for 1 h at RT, washed with 0.1% Tween-20 in PBS (PBS-T), and then further incubated with the anti-CHIKV antibodies (10 µg/mL) for 1 h at 37 °C, followed by further incubation with HRP-conjugated Protein A (1:8000, Invitrogen, Thermo Fisher Scientific) for 1 h at 37 °C. The positive controls: VEEV, hyperimmune serum 1:10 (RIH Research); DENV-1, monoclonal antibody D2-1F1-3 1:10 (Biotem, Le Rivier d’Apprieu, France); DENV-2 and ZIKV, mouse Anti-flavivirus Envelope Protein Antibody (4G2, 1:10; Native Antigen Company, UK) were included. All measurements were made at least three times. Statistical analysis was performed by one-way ANOVA followed by post hoc Tukeys’s multiple comparisons test.

### Western blot

CHIKV (2 µg/mL) were separated by 10% SDS-PAGE and transferred to nitrocellulose membranes (0.2 μm; Biorad, CA, USA) at 25 V and 1.3 A. After blocking with 3% skim milk in PBS-T, the membranes were incubated with the anti-CHIKV antibodies (10 µg/mL) overnight at room temperature. Afterwards, the membranes were washed three times with PBS-T and further incubated with goat anti-human IgG Fc-HRP (1:5,000, Abcam, US) for 1 h. The immunoreactive bands were detected using ECL Western Blotting Substrate (Pierce, Thermo Scientific) and visualized in a Chemidoc^™^ MP System.

### Detection of CHIKV by a sandwich ELISA

Nunc MaxiSorp™ flat-bottom plates were coated with 100 µL of 10 µg/mL of the C7 antibody in carbonate buffer pH 9.4 overnight at 4 °C and blocked with 3% MPBS for 60 min at 37 °C. Serial dilutions of the CHIKV purified and UV-inactivated extract, starting at a concentration of 40 µg/mL, were incubated for 1 h at 37 °C. After washing the non-bond CHIKV, biotinylated 4N12 antibody (0.1 µg/mL) was added and incubated at 37 °C for 1 h. The assay was revealed with Streptavidin conjugated to HRP (1:1,000; Invitrogen Cat. No. S911) and TMB Substrate Reagent Set (BD OptEIA, BD Biosciences; Cat No. 555214). The reaction was stopped with 1 M phosphoric acid, and the absorbance was read at 450/570 nm by using automated BioTek´s 2 microplate reader.

### Number of virions in the purified CHIKV preparation

To estimate the number of CHIKV particles per mL in the UV-inactivated extract, we first determined the mass of E2 protein in our preparation as follows. Three volumes: 10, 5 and 2 µL of the CHIKV stock at a total protein concentration of 691 µg/mL 2D-Quant Kit (Sigma Aldrich) were loaded into an SDS-PAGE gel and ran side-by-side with a standard of known mass (0.5 µg of BSA). The density of the E2 bands was quantified by comparison with the density of the BSA standard. The concentration of E2 was estimated in 36 µg/mL, which is 5.15% of the CHIKV UV-inactivated stock. The number of CHIKV particles per mL (CHIKV/mL) in 40 µg/mL of our CHIKV preparation was calculated in 1.2 × 10^10^ CHIKV/mL as follows: # of molecules of CHIKV/mL = {[(40 µg/mL ×  0.0515)/ 240)] / 46,000 g/mol} ×   6.022 × 10^23^ number of particles/mol x 10^− 6^; where 40 µg/mL is the concentration of the CHIKV UV-inactivated in the starting dilution (see above); 0.0515 is the fraction of E2 protein; 46,000 g/mol is the molecular weight of E2; 240 is number of E2 copies per CHIKV particle [28]; 6.022 × 10^23^ number of particles/mol is Avogadro’s number; and 10^− 6^ is a factor to convert µmol into mol.

### Statistical analysis

Data are shown as the average ± standard error of the mean (SEM). To determine the affinity constants by direct binding ELISA, the results were adjusted following the 4-Parameter Logistic (4PL) curve model and each experiment was carried out at least three times with independent samples. Data were analyzed by one-way ANOVA followed by Tukey’s multiple comparisons tests for the cross-reactivity experiments. Differences were considered statistically significant with a *p* value < 0.05. Statistical analyses were performed in Prism 9.2.0 (Graphpad Software, LLC).

## Results

### CHIKV-033 purification and characterization

A detailed characterization of CHIKV-033, including sequencing of its whole genome, will be published elsewhere (Rosalia Lira et al. personal communication). In this work, to identify the phylogenic origin of this CHIKV strain, we compared the whole genome and E2 of CHIKV-033 with the sequences of fifty-four CHIKV strains including 10 sequences from WA, 18 from Asian, five from ECSA and 26 from IOL lineages (Additional file 1: Fig. S1). The phylogenetic trees of both the whole genome and E2 indicated that the CHIKV-033 belongs to the Asian lineage.

**Fig. 1:**
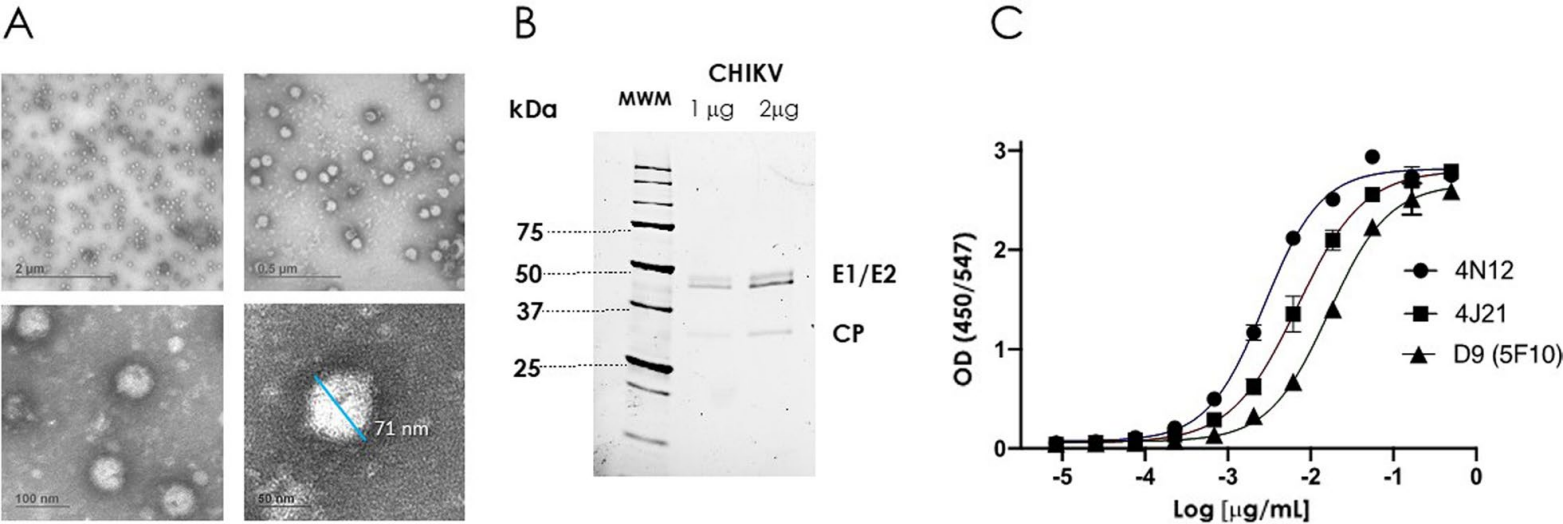
Identity and functional analysis of the CHIKV-033 isolate. **A** Representative electron micrographs of negative staining of CHIKV-033 particles purified by sucrose gradient. Images were captured using the Zeiss Libra 120 transmission electron microscope at 80 kV (Scale bars, 2 μm, 0.5 μm, 100 nm and 50 nm). **B** SDS-PAGE pattern of purified CHIK-033 virions. MiniProtean Stain Free gels (Biorad) showed the typical pattern of the spike proteins, i.e., E1 at ∼48 kDa, E2 at ∼46 kDa and capsid protein (CP) at ∼30 kDa. Precision Plus Protein Unstained Standards (Biorad) was used like molecular weight marker. **C** Direct ELISA of the anti-CHIKV 4N12, 4J21 and D9 (5F10) control antibodies. Each experiment was carried out at least three times with independent samples. Representative numbers are presented as average ± standard error of the mean (SEM)

Electron microscopy of CHIKV-033 purified virions, SDS-PAGE and direct ELISA using three known anti-CHIKV antibodies [4N12, 4J21 and D9 (5F10)] is shown in Fig. 1. The purified viral particles had the proper size (~ 70 nm) and the spherical shape previously described for CHIKV [29, 30] (Fig. 1A). The SDS-PAGE showed the typical pattern of the spike proteins, i.e., E1 ∼48 kDa, E2 ∼46 kDa and capsid protein (CP) ∼30 kDa [31] (Fig. 1B). In addition, the UV-inactivated CHIKV particles were specifically recognized by the three well-characterized anti-CHIKV antibodies (Fig. 1C), indicating that the CHIKV capsid proteins were properly folded after the UV-inactivation process.

### Panning with viral particles and screening for CHIKV specific clones

Having qualified CHIKV-033 isolate, the UV-inactivated purified particles were used as selectors to isolate human antibodies from ALTHEA Gold Libraries™ [24]. The panning was performed in solid phase, e.g., ELISA plates. The screening for specific anti-CHIKV antibody fragments was conducted following two strategies: (1) direct binding to CHIKV particles and BSA, the latter as specificity control; and (2) NGS and data mining.

Direct ELISA of 88 colonies chosen at random from the third round (R3) of panning showed eight positive and specific IPTG-induced supernatants (8/88; 10 %). Sanger sequencing of these eight clones resulted in four unique sequences, called C10, C7, A11 and F2, giving a hit rate of 4.5 %. This hit rate compared well with commonly observed hit rates obtained with other targets and diverse phage display platforms [25]. C10 was found 4 times, whereas, C7 was found twice, and A11 and F2 were found once. To enrich for additional specific scFvs, a fourth round (R4) of panning was performed. Out of 83 IPTG-induced supernatants tested in ELISA, 68 (68/85; Analysis 82%) resulted positive and specific. C10 represented 60% of the positive clones, whereas C7 and A11 showed up 32 % and 8%, respectively. F2 was not seen in R4 and was not processed further.

The NGS statistics are shown in Additional file 1. Over 1.5 million reads were processed from round 2 (R2) to R4. The frequencies and enrichment factors of the HCDR3 sequences of C7, C10 and A11 are shown in Table 1. These clones contributed close to 80% of all the unique sequences in R4. As expected, an enrichment in the frequency of C10, C7 and A11 HCDR3 sequences occurred as the rounds of selection proceeded. Consistent with the ELISA-based screening, C10 was enriched over three orders of magnitude in R4 with respect to R2, whereas C7 was enriched two orders of magnitude and A11 increased its frequency 20-fold from R2 to R4. Additional 6 HCDR3 amino acid sequences not seen in the ELISA-based screening were also enriched within the 50 most frequent HCDR3 sequences in R4. The scFvs having these HCDR3 sequences were amplified using specific primers matching the HCDR3 nucleotide sequences and cloned as human IgG1/kappa antibodies.

**Table 1:** Frequency per round of panning of the antibodies selected via ELISA-based screening and Enrichment Factor (EF)

	R2 (%)	R3 (%)	R4 (%)	EF (3/2)	EF (4/2)
C10	0.04	15.97	49.07	399	1,127
C7	0.04	3.25	26.23	81	656
A11	0.09	1.43	1.75	16	19
Total	0.17	20.65	77.05	–	–

The frequency was calculated as the number of counts of the same HCDR3 sequence divided by the total number of counts per round of panning. EF is the ratio of frequencies of the R3 or R4 with respect to R2, (3/2) and (4/2), respectively

### Characterization of the anti-CHIKV antibodies

Four out the six antibodies coming out of the NGS screening did not show significant binding and were excluded from this study. The characterization of the remining five antibodies isolated from ALTHEA Gold Libraries™ is summarized in Table 2. As a reference, the three control antibodies: 4N12, 4J12 and D9(5F10) are also included in the Table.

**Table 2:** Summary of the characteristics of the anti-CHIKV antibodies obtained from ALTHEA Gold Libraries™

Name	Library^a^	HCDR3 (length)^b^	EC_50_ scFv^c^	K_D_ (nM) scFv	EC_50_ IgG^c^	K_D_ (nM) IgG	Tm (ºC) IgG^d^	Yield IgG (mg/L)
C7	SL2	8	0.534	17.76	0.0160	0.090	69(78)	59.8
C10	SL1	7	1.349	44.9	0.846	5.060	69(86)	59.4
A11	SL2	8	1.479	49	1.509	8.570	69	44.3
NGS2	SL2	17	ND	ND	63.530	423.010	70(81)	7.9
NGS4	SL2	14	ND	ND	0.714	4.630	70	9.9
4N12	ND	14	ND	ND	0.00243	0.016	61	25.1
4J21	ND	18	ND	ND	0.00689	0.046	65	14.1
D9 (5F10)	ND	ND	ND	ND	0.01651	0.11	ND	ND

ND, not determined

^a^SL1 and SL2 are included in ALTHEA Gold Libraries™

^b^The HCDR3 length was defined using Kabat numbering

^c^The half maximal effective concentration (EC50) was determined by quantitative ELISA (see Fig. 3)

^d^The melting temperature (Tm) was determined by Protein thermal shift assay (see Additional file 1: Fig. S3)

One out the five antibodies was isolated from the library called SL1 (second column of Table 2). The other four were obtained from SL2. The SL1 library has a V_L_ scaffold assembled with the human IGKV3-20*01 germline gene combined with the human IGJV4*01 joining region. The SL2 library was made with a V_L_ built with the human IGKV4-01*01 germline gene also combined with the IGJV4*01 germline gene. Both libraries have a universal V_H_ scaffold, which was partially built with the human IGHV3-23*01 germline gene. Therefore, all antibodies but C10 have the same V_H_:V_L_ combination (VH3-23:VL3-20), whereas, C10 has the VH3-23:VL4-01 combination. Interestingly, all the antibodies obtained in the ELISA-based screening have a relatively short HCDR3 sequence (seven to eight residues, third column of Table 2) as compared to the two NGS-obtained antibodies, which have a relatively long HCDR3 (fourteen and seventeen residues).

The integrity of the purified anti-CHIKV antibodies was verified by SDS-PAGE and analytical SEC (Additional file 1: Fig. S2). The expression yield in HEK 293 cells (last column of Table 2) of the antibodies selected based on ELISA was five-fold or higher than that of the NGS antibodies. The expression yield of C7, C10 and A11 was also higher than the control antibodies (4N12 and 4J21). A higher expression yield of C7, C10 and A11 may explain because, although both ELISA-based and the NGS-identified antibodies were enriched during the panning, only C7, C10 and A11 were identified in the ELISA-based screening.

The Tm of the antibodies is also reported in Table 2 (eighth column, see also Additional file 1: Fig. S3). All the five antibodies obtained in this work have Tm values ≥ 69 °C, with C10, C7 and NGS2 having two unfolding transitions. The second transition is close or above 80 °C. In contrast, the control antibodies (4N12 and 4J21) have a single transition at 61 °C or 65 °C, respectively. The control antibodies were isolated from a CHIKV convalescent patient, although they present high affinity, they did not exhibit high thermostability.

**Fig. 2:**
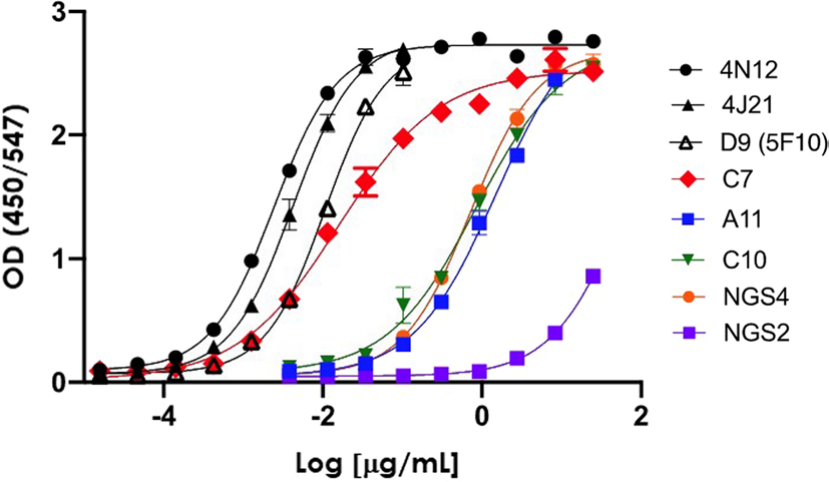
Direct binding ELISA of the anti-CHIKV antibodies isolated from ALTHEA Gold Libraries^™^. The control antibodies were assayed side-by-side with our antibodies. Each experiment was carried out at least three times with independent samples, and representative numbers are presented as average ± SEM. The curves were adjusted to a 4-Parameter Logistic (4PL) curve to determine the EC_50_ and apparent affinity values

Binding of the antibodies to UV-inactivated CHIKV particles—determined as the EC_50_ of direct ELISA curves—is reported in the seventh column of Table 2. The dose-response curves are shown in Fig. 2. Conversion from scFv to IgG1 improved binding to the CHIKV particles by C7, C10 and A11 antibodies (compare the fifth and seventh column, Table 2). One of the two antibodies isolated by NGS, NGS4, showed a similar binding profile than two of the antibodies identified by ELISA-based screening: C10 and A11. NGS2 bound CHIKV particles poorly and was not further characterized.

C7 happened to be the highest affinity antibody isolated from ALTHEA Gold Libraries™, with a binding profile close to the control antibodies D9(5F10) and 4J21. We estimated 0.016 and 0.045 nM for 4N12 and 4J21, respectively. C7 gave an apparent affinity in the ELISA of 0.09 nM which is twofold the value measured for 4N12. The K_D_ values reported for the control antibodies, 4N12 and 4J21, when measured by BIAcore using the recombinant CHIKV capsid proteins are 0.95 and 0.54 nM, respectively [13]. Therefore, we can safely assume that the K_D_ of C7 if measured in the BIACore with recombinant CHIKV proteins would be in the single nM or sub-nM range. The difference with the affinity measured by ELISA and BIAcore could be due to a number of factors including: (1) mutations of CHIKV-033 affecting binding to these antibodies, (2) configuration and arrangement of the E2 protein in the UV-inactivated CHIKV with respect to isolated recombinant proteins, (3) isotype of the antibodies—notice that we used different isotypes than the original publication [13] and (4) assay conditions.

### Cross-reactivity with VEEV, DENV and ZIKV

**Fig. 3:**
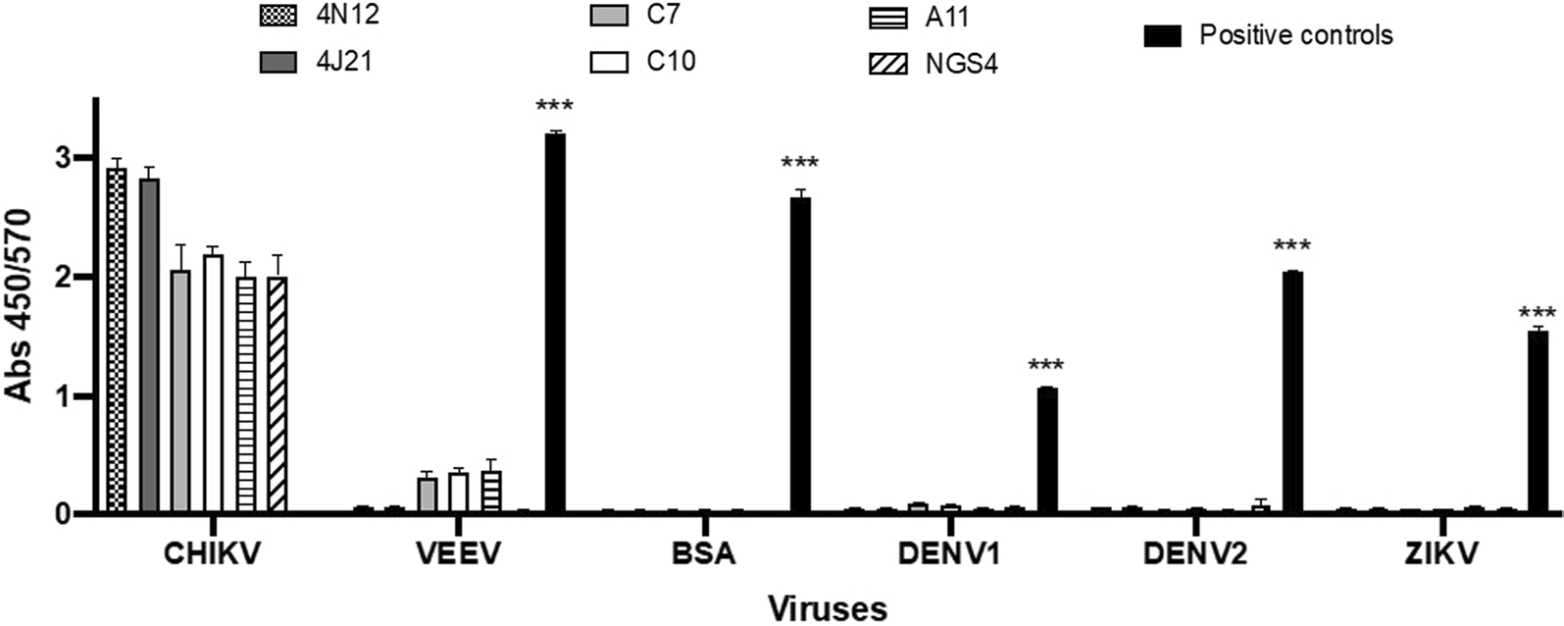
Specificity of the anti-CHIKV human antibodies. Inactivated arbovirus (CHIKV, VEEV, DENV-1, DENV-2, and ZIKV) were coated onto ELISA plates. After the blocking, all the antibodies were used at the same concentration and detected with HRP-conjugated Protein A and enhanced by chemiluminescence (ECL) substrate. For the recognition of each virus, a hyperimmune serum or monoclonal antibodies specific for each virus were used as positive controls (see details in "Method" section). BSA was used as negative control. Corrected OD values are shown. All measurements were caried out in triplicate. Data are shown as average ± standard error of the mean (SEM). Statistical analysis was performed by one-way ANOVA followed by post hoc Tukeys’s multiple comparisons test. *** indicates *p* < 0.001

Cross-reactivity of C7, C10, A11 and NGS4 with VEEV in shown in Fig. 3. C7, C10 and A11 had a slight cross-reactivity with VEEV, whereas NGS4 did not show cross-reactivity. Interestingly, cross-reactive immune response with VEEV after immunization with CHIKV has been reported [32]. None of the antibodies showed cross-reactivity with DENV1, DENV2 and ZIKV.

### Reactivity of the antibodies with CHIKV proteins

**Fig. 4:**
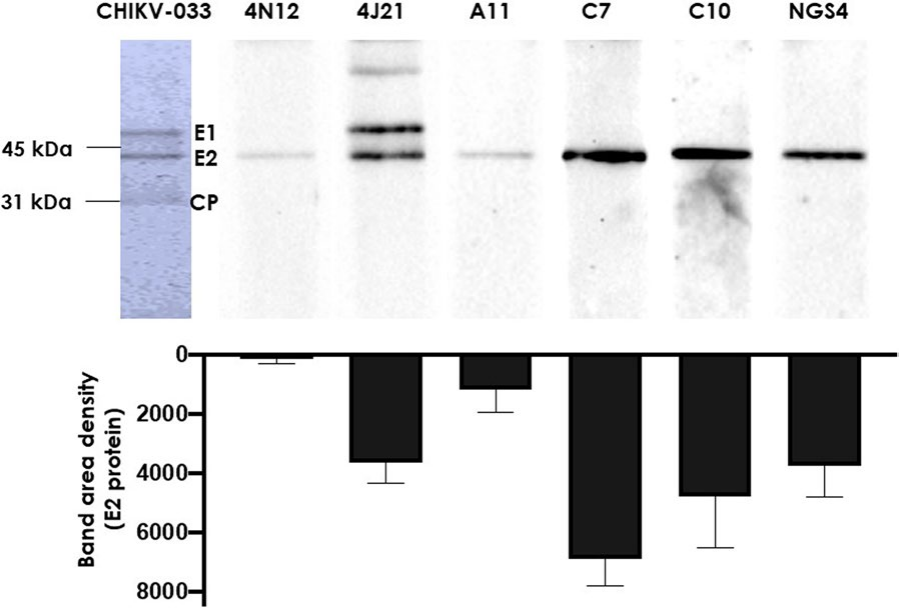
Representative western blot analysis of the antibodies isolated in this work. Inactivated CHIKV-033 proteins were separated by 10% SDS-PAGE and transferred onto nitrocellulose membranes. The blocked membranes were probed with the anti-CHIKV human antibodies from ALTHEA Gold Libraries^™^, followed by incubation with HRP-conjugated anti-human IgG. The 4N12 and 4J21 antibodies were used for comparison. In the lower panel, the E2 band densitometry values recognized by the anti-CHIKV human antibodies are shown. All measurements were made at least in duplicate. Data are shown as average ± SEM

To identify the CHIKV proteins recognized by C7, C10, A11 and NGS4, a western blot with the UV-inactivated CHIKV particles was performed (Fig. 4 above). All the antibodies recognized specifically the structural protein E2, with C7 showing a band of higher intensity than that of C10, NGS4 and A11 (Fig. 4 below). A higher E2 binding by C7 with respect to the other antibodies correlates with the binding profile obtained by ELISA except for A11 (see above). A11 showed a similar ELISA binding profile than C10 and NGS4 but barely recognized CHIKV in the western blot. Likewise, 4N12 gave a weak signal in the western blot, despite being the highest affinity antibody by ELISA. This result suggested that 4N12 (and perhaps A11) recognizes a conformational epitope on E2, whereas C7, C10 and NGS4 bound continuous epitopes.

### Detection of CHIKV particles using a sandwich ELISA

Based on the above results and the binding profile of the antibodies (Fig. 2), we decided to generate a sandwich ELISA with C7 and 4N12 to explore the potential of this assay in quantifying CHIKV particles in biological samples. To this end, both antibodies were biotinylated and used in alternative formats, i.e., C7 as capture reagent and 4N12 as reporter, and vice versa. The format with C7 as capture reagent and 4N12 as reporter gave the higher dynamic range and sensitivity (data not shown) and was thus used in the subsequent experiments.

**Fig. 5:**
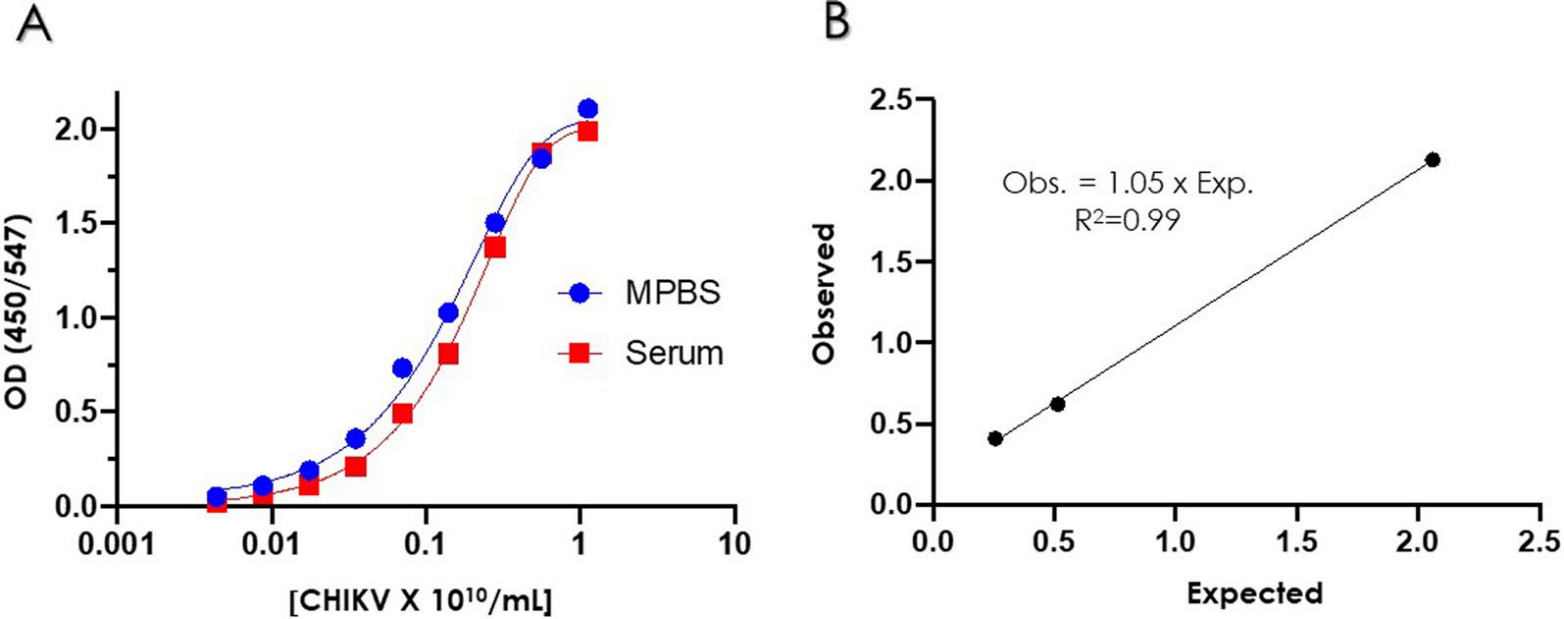
**A** Sandwich ELISA using C7 as capture antibody and biotinylated 4N12 as reporter. The dose/response curves were prepared with 1:2 serial dilutions of the UV-inactivated CHIKV preparation in CHIKV-free serum or assay buffer. The concentration of CHIKV is expressed in terms of CHIKV particles per mL, based on the concentration of E2 as described in detail in the "Method" section. **B** Recovery assay showing samples of CHIKV spiked at known concentration in serum and measured in the sandwich ELISA

To determine how serum affected binding of the antibodies to CHIKV particles, serial dilutions of the UV-inactivated and purified CHIKV preparation in both 1:2 serum and assay buffer (MPBS) were prepared and assayed in the sandwich ELISA. Both dilution matrixes gave similar curves (Fig. 5A; Additional file 2). The minimum concentration of CHIKV particles per mL detected was 1.9 × 10^8^, estimated as twofold the assay background. The curves plateaued at 1.8 × 10^10^ CHIKV/mL, for a dynamical range of 1.9 × 10^8^–1.8 × 10^10^ CHIKV/mL.

To determine the recovery of CHIKV particles in serum we spiked known concentrations of the UV-inactivated and purified CHIKV preparation in CHIKV-free serum and compared the expected and measured value. The R^2^ was 0.99 and a slope of the regression curve was close to one (Fig. 5B), indicating a good recovering of the CHIKV in serum.

## Discussion

In the previous sections we reported the characterization of a panel of anti-CHIKV antibodies. Our strategy to obtain these antibodies differed from other methods currently described to isolate CHIKV antibodies [13, 21]. These methods have used VLPs or recombinant capsid proteins as immunogens to obtain polyclonal or monoclonal antibodies from diverse species. In other instances, VLPs or recombinant proteins have been employed as selectors combined with immune libraries or B-cell sorting methods as source of antibodies. Here, we used UV-inactivated CHIKV particles as selectors and ALTHEA Gold Libraries™ [24, 25] as source of antibodies. Different from immune libraries, which are prepared with B-cells isolated from patients infected with a given virus, ALTHEA Gold Libraries™ are general purpose libraries that can be used to obtain antibodies against any given target.

After three rounds of panning with UV-inactivated CHIKV particles, the screening by ELISA of a relatively small number of clones (< 100) yielded three unique anti-CHIKV antibodies. Two additional antibodies were discovered by a complementary screening strategy based on NGS and data mining. Interestingly, the antibodies selected by ELISA and those obtained by NGS differed in several respects, including expression and cross-reactivity with VEEV, a virus closely related CHIKV. At least two different epitopes on the CHIKV E2 protein were recognized by these antibodies. Most of the antibodies recognized linear epitopes on E2, whereas, one antibody, A11, seemed to bind a non-linear epitope. The best antibody, C7, showed an affinity in the single digit nM or sub-nM range, comparable to some of the best anti-CHIKV antibodies described to date [13, 21].

To explore the value of C7 as substrate for development of CHIKV detection methods, a sandwich ELISA with C7 and 4N12 was performed. Both antibodies bind E2 but via different epitopes; C7 binds a linear epitope, whereas 4N12 binds a conformational one. The dynamic range of the sandwich ELISA was 0.2–40 mg/mL of UV-inactivated and purified CHIKV preparation. Based on the concentration of E2 in the CHIKV preparation, we estimated that this range of concentrations translates into 10^8^–10^10^ CHIKV particles per mL. Although this estimation rests on a number of assumptions, some authors [33] have reported that the CHIKV load during the acute phase of the viremia is 10^9^–10^12^ CHIKV/mL of blood. Other authors [34] have measured concentrations of CHIKV in samples drawn on day 0 of the infection around 10^9^ RNA copies/mL, with viral loads decreasing over time. Yet another report [35] has determined that the median viral load ranges from 2.9 × 10^4^ to 1.6 × 10^8^ plaque forming units (PFU) per mL during days 1 through 4 of the viremia period. Considering that PFU underestimate the actual number of viral particles by one or two orders of magnitude due to variations of in the infectivity of the virus, values of 10^9^ CHIKV/mL or RNA copies/mL or 1.6 × 10^8^ PFU/mL fall well within the 10^8^–10^10^ CHIKV/mL dynamical range of the sandwich ELISA. Therefore, although optimizable, this assay could serve as the basis for detection of meaningful CHIKV loads during the acute phase of the CHIKV infection.

It should be noted that our antibodies showed superior expression, highly pure preparations after a single purification step and higher stability than the best known CHIKV antibodies [13, 21]. These favorable pharmaceutical properties could translate in diagnostic tests produced at lower costs, with longer shelf stability and higher robustness. Nevertheless, these antibodies have not been tested in independent clinical samples from CHIKV RNA-positive patients or from different CHIKV strains, which limit their immediate uses. This information will be essential to future investigations, above all, in the case of new CHIKV infections appear.

## Conclusions

We report here the discovery and characterization of new anti-CHIKV human antibodies. These antibodies showed specificity and high affinity to the CHIKV virions, with a superior expression, highly pure preparations after a single-step purification and high stability. These characteristics make the antibodies described here valuable tools in developing cost-effective assays for early stages detection and disease control of the CHIKV infection in future outbreaks.

## Supplementary Information

**Additional file 1.** Supplementary material.

**Additional file 2.** Uncropped figures.

**Additional file 3.** Principal characteristics in the sequence of the heavy and ligth variable chains of the anti-Chikungunya antibodies.

**Additional file 4.** Nucleotide sequence of 033 isolate Chikungunya virus in fasta format.

## Data Availability

The datasets generated and/or analysed during the current study are available in the GenBank repository, [Accession numbers: OK247726, OK247727, OK247728, OK247729, OK247730, OK247731, OK247732, OK247733, OK247734, OK247735] (Additional files 3, 4).

## Abbreviations

CHIKV: Chikungunya virus
ELISA: Enzyme-Linked ImmunoSorbent Assay
IGHV: Immunoglobulin heavy-chain variable
IGKV: Immunoglobulin kappa-chain variable
HCDR3: Third complementary determining region of heavy-chain variable

## References

[1] ROSS RW (1956). The Newala epidemic. III. The virus: isolation, pathogenic properties and relationship to the epidemic. J Hyg (Lond).

[2] Schneider AB (2019). Updated phylogeny of Chikungunya virus suggests lineage-specific RNA architecture. Viruses.

[3] Johansson MA (2015). Chikungunya on the move. Trends Parasitol.

[4] Rivera-Ávila RC (2014). Chikungunya fever in Mexico: confirmed case and notes on the epidemiologic response. Salud Publica Mex.

[5] Kautz TF (2015). Chikungunya virus as cause of febrile illness outbreak, Chiapas, Mexico, 2014. Emerg Infect Dis.

[6] Díaz-González EE (2015). First report of *Aedes aegypti* transmission of Chikungunya virus in the Americas. Am J Trop Med Hyg.

[7] Nava-Frías M (2016). Chikungunya fever: current status in Mexico. Bol Med Hosp Infant Mex.

[8] Wang P, Zhang R (2019). Chikungunya virus and (Re-) emerging alphaviruses. Viruses.

[9] Nsoesie EO (2016). Global distribution and environmental suitability for chikungunya virus, 1952 to 2015. Euro Surveill.

[10] Volk SM (2010). Genome-scale phylogenetic analyses of chikungunya virus reveal independent emergences of recent epidemics and various evolutionary rates. J Virol.

[11] Tsetsarkin KA (2011). Chikungunya virus: evolution and genetic determinants of emergence. Curr Opin Virol.

[12] Okabayashi T (2015). Detection of chikungunya virus antigen by a novel rapid immunochromatographic test. J Clin Microbiol.

[13] Smith SA (2015). Isolation and characterization of broad and ultrapotent human monoclonal antibodies with therapeutic activity against Chikungunya virus. Cell Host Microbe.

[14] Powers AM (2018). Vaccine and therapeutic options to control Chikungunya virus. Clin Microbiol Rev.

[15] Langsjoen RM (2018). Chikungunya virus strains show lineage-specific variations in virulence and cross-protective ability in murine and nonhuman primate models. mBio.

[16] Pongsiri P (2012). Multiplex real-time RT-PCR for detecting chikungunya virus and dengue virus. Asian Pac J Trop Med.

[17] Mourya DT, Mishra AC (2006). Chikungunya fever. Lancet.

[18] Shukla J (2009). Development and evaluation of antigen capture ELISA for early clinical diagnosis of chikungunya. Diagn Microbiol Infect Dis.

[19] Okabayashi T (2016). Correction for Okabayashi et al., Detection of Chikungunya Virus Antigen by a Novel Rapid Immunochromatographic Test. J Clin Microbiol.

[20] Tuekprakhon A (2018). Broad-spectrum monoclonal antibodies against chikungunya virus structural proteins: Promising candidates for antibody-based rapid diagnostic test development. PLoS One.

[21] Warter L (2011). Chikungunya virus envelope-specific human monoclonal antibodies with broad neutralization potency. J Immunol.

[22] Jain J (2018). Evaluation of an immunochromatography rapid diagnosis kit for detection of chikungunya virus antigen in India, a dengue-endemic country. Virol J.

[23] Huits R (2018). Diagnostic accuracy of a rapid E1-antigen test for chikungunya virus infection in a reference setting. Clin Microbiol Infect.

[24] Valadon P (2019). ALTHEA Gold Libraries™: antibody libraries for therapeutic antibody discovery. MAbs.

[25] Almagro JC (2019). Phage display libraries for antibody therapeutic discovery and development. Antibodies (Basel).

[26] Almagro JC (2004). Identification of differences in the specificity-determining residues of antibodies that recognize antigens of different size: implications for the rational design of antibody repertoires. J Mol Recognit.

[27] Raghunathan G (2012). Antigen-binding site anatomy and somatic mutations in antibodies that recognize different types of antigens. J Mol Recognit.

[28] Mukhopadhyay S (2006). Mapping the structure and function of the E1 and E2 glycoproteins in alphaviruses. Structure.

[29] Sun S (2013). Structural analyses at pseudo atomic resolution of Chikungunya virus and antibodies show mechanisms of neutralization. Elife.

[30] Simizu B (1984). Structural proteins of Chikungunya virus. J Virol.

[31] Silva LA, Dermody TS (2017). Chikungunya virus: epidemiology, replication, disease mechanisms, and prospective intervention strategies. J Clin Invest.

[32] McClain DJ (1998). Immunologic interference from sequential administration of live attenuated alphavirus vaccines. J Infect Dis.

[33] Das T (2010). Chikungunya fever: CNS infection and pathologies of a re-emerging arbovirus. Prog Neurobiol.

[34] Panning M (2008). Chikungunya fever in travelers returning to Europe from the Indian Ocean region, 2006. Emerg Infect Dis.

[35] Appassakij H (2013). Viremic profiles in asymptomatic and symptomatic chikungunya fever: a blood transfusion threat?. Transfusion.

