# Supplementary material for: Isolation and characterization of high affinity and highly stable anti-Chikungunya virus antibodies using ALTHEA Gold Libraries™
Source: BMC Infect Dis. 2021 Oct 30;21:1121. doi: 10.1186/s12879-021-06717-0 (PMC8556770; doi:10.1186/s12879-021-06717-0)
Supplement: Supplementary file 1 — Additional file 1. Supplementary material. [file 12879_2021_6717_MOESM1_ESM.docx]

**Additional File 1**

**Supplementary Material**

**Isolation and characterization of high affinity and highly stable anti-Chikungunya virus antibodies using ALTHEA Gold Libraries™**

M. Pedraza-Escalona1.3; O. Guzmán-Bringas2,3; I. Arrieta-Oliva 2,3; K. Gómez- Castellano2,3; J. Salinas-Trujano2,3; J. Torres-Flores2; J.C. Muñoz-Herrera2,3; R. Camacho-Sandoval 2,3; P. Contreras-Pineda2,3; R. Chacón-Salinas2,3,4; SM. Pérez-Tapia2,3,4; JC. Almagro2,3,5*.

1CONACyT-Unidad de Desarrollo e Investigación en Bioprocesos (UDIBI), Escuela Nacional de Ciencias Biológicas, Instituto Politécnico Nacional, México

2Unidad de Desarrollo e Investigación en Bioprocesos (UDIBI), Escuela Nacional de Ciencias Biológicas, Instituto Politécnico Nacional, México.

3Laboratorio Nacional para Servicios Especializados de Investigación, Desarrollo e Innovación (I+D+i) para Farmoquímicos y Biotecnológicos, LANSEIDI-FarBiotec- CONACyT, México.

4Departamento de Inmunología, Escuela Nacional de Ciencias Biológicas, Instituto Politécnico Nacional (ENCB-IPN), Mexico City, Mexico

5GlobalBio, Inc. 320 Concord Ave, Cambridge, MA 02138, USA.

**CHIKV-033 Isolate Complete Genome Sequencing**

Viral RNA of the CHIKV was extracted by using a QIAamp viral RNA minikit (Qiagen, Germany) and the sequencing library was constructed using the TruSeq Stranded mRNA Library Preparation Kit (Illumina Cat No. 20020594) and was sequenced by Illumina NextSeq™ 550 system. The raw sequence reads (10,970,848 reads) were processed using Geneious R10 version 10.2.6 software. Complete genome sequence assembly was achieved by mapping to a reference CHIKV genome sequence (strain S27, GenBank accession number AF369024.2) that included 5′ and 3′ untranslated terminal sequences, using default parameters and the low-sensitivity setting.

**Additional file 1: Figure S1.** Phylogenetic tree from the whole genomes **(left)** and E2 protein **(right)** inferred for 59 CHIKV genome sequences from the West Africa, Asian and East/Central/South Africa-Indian Ocean lineages (Pyke AT, et al., 2020. Genome sequences of chikungunya virus strains from Bangladesh and Thailand. Microbiol Resour Announc 9:e01452-19.https://doi.org/10.1128/MRA.01452-19.). The sequence of CHIKV-033 isolate is marked with a green dot. Branch lengths are proportional to the number of substitutions per site. Numbers next to the accession number indicate the bootstrap values. Multiple sequence alignment was performed using MUSCLE (https://www.ebi.ac.uk/Tools/msa/muscle/). The tree was built with Simple Phylogeny (<https://www.ebi.ac.uk/Tools/phylogeny/simple_phylogeny/>) using the neighbor-joining method.

**Quality Control of the antibodies isolated from ALTHEA Gold Libraries™**

Three hundred mL of HEK 293 were co-transfected with the plasmids having the heavy and light chains. After four days of incubation, the supernatants were purified using Protein A MabSelect SuRe column (5 mL, GE Healthcare). The IgGs were captured in 20 mM Phosphate buffer, 150 mM NaCl, pH 7.4 and eluted with 20 mM citrate buffer pH 3.5. The monomeric content of the purified IgGs was estimated by UPLC BEH200 150 mm SEC column (Waters) and the integrity was determined by SDS-PAGE.

**Additional file 1: Figure S2. SDS-PAGE (A) and size-exclusion chromatography (SEC) profile (B) of the anti-CHIKV antibodies after Protein A purification.** One band from ~150 kDa (complete IgG) is observed under non-reducing (NR) conditions. Two bands, ~50 kDa (heavy chains) and ~25kDa (light chains), were observed under reducing (R) conditions. Any kD™ Mini-PROTEAN^®^ TGX Stain-Free™ Protein Gels (Biorad) and Precision Plus Protein Unstained Standards (molecular weight marker, Biorad) were used. The elution volumes of SEC molecular weight standards are shown in arrows: a) thyroglobulin (670 kDa), b) γ-globulin (158 kDa), c)ovalbumin (44 kDa) and d)myoglobin (17 kDa). The MWs from anti-CHIKV antibodies ranged from 154 to 172 kDa.

**Anti-CHIKV antibodies thermal stability assay**

Unfolding transitions were estimated using a fluorescent dye (SYPRO Orange, 480 nm excitation and 610 nm emission). Each antibody (4 μg) was heated from 25 ^o^C to 99 ^o^C at 5 ºC/minute. The Temperature melting (T_m_) was calculated using the first derivative of the fluorescence emission as a function of temperature (dFluorescence/d*T*).

**Additional file 1: Figure S3. Unfolding profiles of the anti-CHIKV antibodies**. The Boltzmann (upper panel) and the first derivative of the fluorescence emission as a function of temperature (dFluorescence/d*T*) (lower panel) melting profiles are shown to each antibody analyzed at a concentration of 4 μg in PBS. The T_m_ is indicated with a vertical arrow in each profile.
