# Supplementary material for: Isolation and characterization of high affinity and highly stable anti-Chikungunya virus antibodies using ALTHEA Gold Libraries™
Source: BMC Infect Dis. 2021 Oct 30;21:1121. doi: 10.1186/s12879-021-06717-0 (PMC8556770; doi:10.1186/s12879-021-06717-0)
Supplement: Supplementary file 2 — Additional file 2. Uncropped figures. [file 12879_2021_6717_MOESM2_ESM.docx]

**Additional file 2. Uncropped figures**

**Additional file 2: Uncropped Figure 1B**. SDS-PAGE pattern of purified CHIK-033 virions. MiniProtean Stain Free gels (Biorad) showed the typical pattern of the spike proteins, i.e., E1 at ~48 kDa, E2 at ~46 kDa and capsid protein (CP) at ~30 kDa. Precision Plus Protein Unstained Standards (Biorad) was used like molecular weight marker.

**Additional file 2: Uncropped Figure 4.** Representative western blot analysis of the antibodies isolated in this work. Inactivated CHIKV-033 proteins were separated by 10% SDS-PAGE and transferred onto nitrocellulose membranes. The blocked membranes were probed with the anti-CHIKV human antibodies from ALTHEA Gold Libraries^™^, followed by incubation with HRP-conjugated anti-human IgG. The 4N12 and 4J21 antibodies were used for comparison. In the lower panel, the E2 band densitometry values recognized by the anti-CHIKV human antibodies are shown. All measurements were made at least in duplicate. Data are shown as average ± SEM.

**Additional file 2: Uncropped Supplementary Figure 2A**. SDS-PAGE of the anti-CHIKV antibodies after Protein A purification. One band from ~150 kDa (complete IgG) is observed under non-reducing (NR) conditions. Two bands, ~50 kDa (heavy chains) and ~25kDa (light chains), were observed under reducing (R) conditions. Any kD™ Mini-PROTEAN^®^ TGX Stain-Free™ Protein Gels (Biorad) and Precision Plus Protein Unstained Standards (molecular weight marker, Biorad) were used. In all the cases Adalimumab was used as control.
